# Impact of timing of radium‑223 administration on the survival of patients with bone metastatic castration‑resistant prostate cancer

**DOI:** 10.3892/mi.2023.98

**Published:** 2023-07-11

**Authors:** Kenji Makita, Yasushi Hamamoto, Hiromitsu Kanzaki, Natsumi Yamashita, Kei Nagasaki, Teruhito Kido, Noriyoshi Miura, Takashi Saika, Katsuyoshi Hashine

**Affiliations:** 1 Department of Radiation Oncology, National Hospital Organization Shikoku Cancer Center, Matsuyama, Ehime 791-0280, Japan; 2 Department of Radiology, Ehime University Graduate School of Medicine, Toon, Ehime 791-0295, Japan; 3 Department of Radiology, Ehime Prefectural Central Hospital, Matsuyama, Ehime 790-0024, Japan; 4 Division of Clinical Biostatistics, Section of Cancer Prevention and Epidemiology, Clinical Research Center, National Hospital Organization Shikoku Cancer Center, Matsuyama, Ehime 791-0280, Japan; 5 Department of Urology, Ehime University Graduate School of Medicine, Toon, Ehime 791-0295, Japan; 6 Department of Urology, National Hospital Organization Shikoku Cancer Center, Matsuyama, Ehime 791-0280, Japan

**Keywords:** radium-223 chloride, optimal timing, survival, metastatic castration-resistant prostate cancer, bone metastasis

## Abstract

The present study aimed to evaluate the optimal timing of radium-223 chloride (Ra-223) administration among patients with bone metastasis from castration-resistant prostate cancer (BmCRPC). Patients, who were diagnosed with BmCRPC and treated with Ra-223 therapy between October, 2016 and January, 2022, were reviewed. The survival time was calculated from the initiation of Ra-223 administration. The time from the diagnosis of BmCRPC to the initiation of Ra-223 administration was identified as a potential prognostic factor. A total of 51 patients were examined in the present study. Ra-223 was administered as the first- and second-line therapy (earlier Ra-223 administration) in 32 patients and as the third- to fifth-line therapy (later Ra-223 administration) in 19 patients. In the multivariate analysis, which considered the potential prognosis, the difference in survival times between patients who received early and late Ra-223 administration was not significant [hazard ratio (HR), 2.67; 95% confidence interval (CI), 0.79-9.07; P=0.11]. By contrast, an incomplete Ra-223 administration (HR, 128.03; 95% CI, 10.59-1548.42; P<0.01) and higher levels of prostate-specific antigen prior to Ra-223 administration (HR, 7.86; 95% CI, 2.7-27.24; P<0.01) were independent factors, significantly associated with a poorer prognosis. The timing of Ra-223 administration did not significantly affect the survival of patients from the initiation of treatment. Further studies are thus required to determine the optimal timing for Ra-223 administration.

## Introduction

Castration-resistant prostate cancer (CRPC) pertains to a prostate malignancy that is not controlled medically or via surgical castration. The treatment of CRPC mainly involves novel pharmaceutical therapy and chemotherapy [abiraterone acetate, enzalutamide, docetaxel (DOC) or cabazitaxel] (1-4). Radium-223 chloride (Ra-223) has emerged as a treatment option for bone metastasis from CRPC (BmCRPC) (5).

Ra-223 accumulates at sites of bone metastases and other regions with a high bone metabolic activity. It emits alpha rays, thereby exerting a tumor-controlling effect (6,7). In an overseas phase III study (ALSYMPCA study, Alpharadin in Symptomatic Prostate Cancer), Ra-223 achieved favorable therapeutic results. It prolonged the overall survival (OS) of patients by 3.6 months and time to bone-related events (SRE) by 5.8 months (5). Thus, Ra-223 was recognized as a viable treatment option that improved the SRE and OS.

Previous studies have identified several clinical factors associated with the poor prognosis of patients with CRPC (8-10). However, the effect of the timing of Ra-223 administration on the survival of the patient remains unclear. Ra-223 is typically administered to patients with progressive disease, despite novel pharmaceutical therapy and chemotherapy. Recent studies have demonstrated that the earlier Ra-223 administration affects the post-treatment prognosis of patients with BmCRPC (11,12). Those studies analyzed the prognostic factors at the time of Ra-223 administration. However, their methods were insufficient to determine the optimal timing of Ra-223 administration as various subsequent treatment options remain available for patients who receive early Ra-223 administration, while the patients who receive later Ra-223 administration have limited subsequent treatment options (11,12). Thus, in the present study, in an aim to address this issue, the association between the survival and timing of Ra-223 administration was analyzed, considering the potential prognosis upon initiating Ra-223 administration.

## Patients and methods

Between October, 2016 and January, 2022, 56 patients were treated with Ra-223 at the National Hospital Organization Shikoku Cancer Center Hospital and Ehime University Hospital (Ehime, Japan). Among these patients, those with small cell carcinoma (n=1), those included in the phase II study of personalized peptide vaccine for DOC-based chemotherapy-resistant CRPC (n=1), and those without follow-up after completing the Ra-223 regimen (n=3) were excluded from the study. Finally, 51 patients (aged 64-90 years; median age, 72 years) were included in the present study. The present retrospective study was approved by the Ethics Committee of Ehime University Hospital and the National Hospital Organization Shikoku Cancer Center (registration no. 2211017). The need for informed consent was waived due to the retrospective nature of the study.

CRPC is defined as prostate cancer with a testosterone level within the castration range (≤50 ng/dl) and a prostate-specific antigen (PSA) level ≥25% from the lowest value, measured at least 4 weeks apart, with an increase of ≥2.0 ng/ml (13).

A total of 48 patients were pathologically diagnosed by needle biopsy, and 3 patients were clinically diagnosed with prostate cancer prior to the initial treatment. Bone metastases were detected via bone scintigraphy in all patients, and osteoplastic bone metastasis was proven via computed tomography. The timing of the Ra-223 administration was decided at the discretion of the physician and institution. Ra-223 was administered at a dose of 50-55 kBq/kg every 4 weeks for up to six cycles.

### Statistical analysis

The survival time of patients following the administration of Ra-223 was calculated from the initiation of Ra-223 administration. The Kaplan-Meier method was used to generate the OS curve. Univariate and multivariate analyses of the survival time after Ra-223 were performed using the Cox proportional hazard model to determine the hazard ratios (HRs), 95% confidence intervals (CIs) and P-values. To evaluate the prognosis upon the initiation of Ra-223 administration, the time from the diagnosis of BmCRPC to the initiation of Ra-223 administration (BmCRPC-Ra223 time) was included in the multivariate analysis. Statistical analyses were performed using JMP software (JMP version 14.3.0; SAS Institute).

## Results

A total of 51 patients with BmCRPC, treated with Ra-223 at the authors' institutions between October, 2016 and January, 2022, were retrospectively reviewed. Laboratory data prior to Ra-223 administration (median, range) were as follows: i) PSA (ng/ml; 15.66, 0.08-616.1); ii) hemoglobin (g/dl; 12.3, 8.1-14.8); iii) platelets (x10^4^/µl; 21.5, 14.3-38.3); iv) alkaline phosphatase (U/l; 239, 57-6047); v) lactate dehydrogenase (U/l; 207, 148-554); vi) neutrophil-to-lymphocyte ratio (2.72, 0.91-17.19); and vii) platelet-to-lymphocyte ratio (0.88, 0.44-6.48). The patient characteristics are presented in Table I.

**Figure 1:**
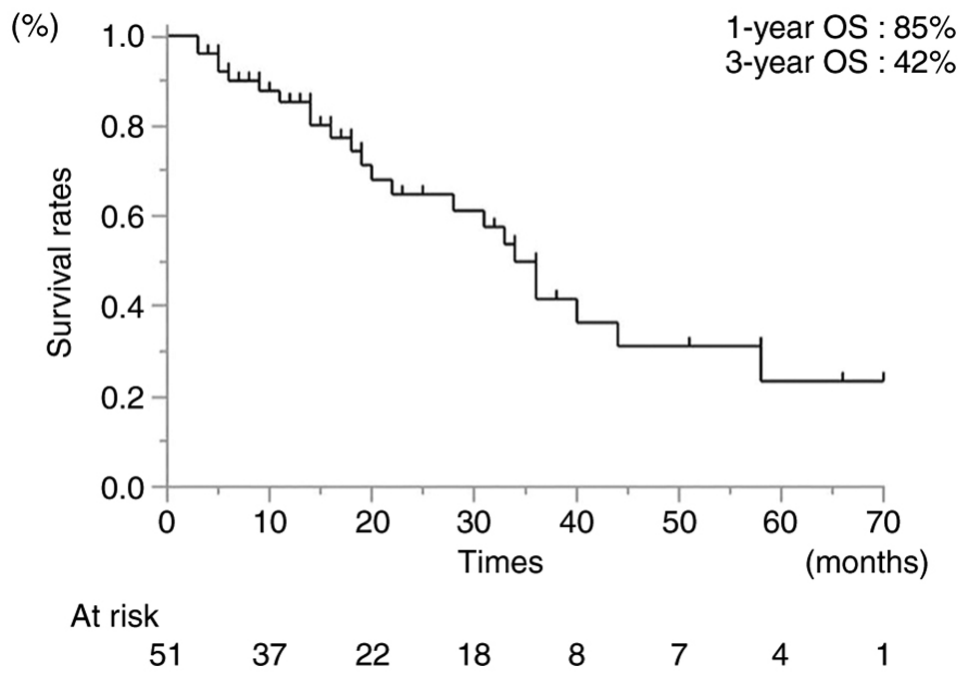
Survival rates following radium-223 chloride therapy. OS, overall survival.

The median follow-up time was 18 months (range, 3-70 months). The 1- and 3-year OS rates following Ra-223 administration were 85 and 42%, respectively (Fig. 1). The 1-year OS rate differed significantly between the patients who received early (first- to second-line) and late (third- to fifth-line) Ra-223 therapy (87 and 74%, respectively; HR, 5.90; 95% CI, 19.7-17.63; P<0.01). In addition, the 1-year OS rates differed significantly between patients who completed or failed to complete the Ra-233 regimen (97 and 37%, respectively; HR, 42.88; 95% CI, 7.91-232.55; P<0.01) (Table II). The reasons for uncompleted Ra-223 regimens were the following: i) Adverse events (myelosuppression, n=1; febrile neutropenia, n=1); ii) the appearance of metastases (liver, n=5; adrenal gland, n=1); iii) other diseases (pneumonia, n=1; sudden death from aortic stenosis, n=1); iv) refusal to undergo Ra-223 treatment (n=1). In patients with a PSA level <15.66 ng/ml and ≥15.66 ng/ml, the 1-year OS rate also differed significantly (100 and 72%, respectively; HR, 6.79; 95% CI, 2.47-18.66; P<0.01). The difference in the 1-year OS rate was also significant between patients with a PS=0 and ≥1 (90 and 78%, respectively; HR, 2.79; 95% CI, 1.21-6.46; P=0.02) (Table II). By contrast, there was no statistically significant difference between patients with a BmCRPC-Ra-223 time <1 year and ≥1 year (HR, 1.78; 95% CI, 0.76-4.18; P=0.19) (Table II).

Although the BmCRPC-Ra-223 time was not a significant factor in the univariate analysis, it was included as a factor in the multivariate analysis as the present study aimed to examine the effects of the potential prognosis following the initiation of Ra-223 administration. Based on the multivariate analysis, including the BmCRPC-Ra-223 time, late Ra-223 therapy (third- to fifth-line) was not significantly associated with an unfavorable prognosis (HR, 2.67; 95% CI, 0.79-9.07; P=0.11) (Table II). By contrast, the incompletion of Ra-223 therapy (HR, 128.03; 95% CI, 10.59-1548.42; P<0.01) and a higher PSA level (≥15.66; HR, 7.86; 95% CI, 2.70-27.24; P<0.01) were independent factors that were significantly associated with a poorer prognosis (Table II). Based on the multivariate analysis excluding the BmCRPC-Ra-223 time, the incompletion of Ra-223 (HR, 154.18; 95% CI, 13.40-1773.89; P<0.01), later-line Ra-233 therapy (third- to fifth-line) (HR, 2.96; 95% CI, 1.00-9.27; P=0.05) and a higher PSA level (≥15.66; HR, 8.23; 95% CI, 2.40-28.36; P<0.01) (Table SI) were significant independent factors, associated with an unfavorable post-treatment prognosis.

## Discussion

In the present study, when BmCRPC-Ra-223 time was analyzed as a prognostic factor, an unfavorable prognosis was associated with an incomplete Ra-223 administration (five cycles or less) and a higher PSA level (≥15.66). By contrast, the timing of Ra-223 administration did not affect the survival of patients with BmCRPC. However, when the BmCRPC-Ra-223 time was not analyzed as a prognostic factor, an unfavorable prognosis was associated with an incomplete Ra-223 administration (five cycles or less), later Ra-233 therapy (third- to fifth-line) and a higher PSA level (≥15.66).

Ra-223 treatment has been shown to prolong the OS rate of patients and improved their quality of life (5). Although the European Medicines Agency (EMA) recommends the administration of Ra-223 to patients who have already received at least two previous treatments (14), the optimal timing of Ra-223 administration in patients with BmCRPC remains controversial. Previous studies have suggested that an early Ra-223 administration in patients with BmCRPC positively affects their survival (7,8,10). However, these studies may have been biased as the timing of Ra-223 administration likely affected the prognosis. Patients who received Ra-223 earlier had longer life expectancies, while those who received Ra-223 later likely had shorter life expectancies. In the present study, when the BmCRPC-Ra-223 time was not evaluated as a prognostic factor, early Ra-223 administration significantly prolonged survival. However, when the BmCRPC-Ra-223 time was assessed as a prognostic factor, it had a smaller impact on survival. Based on these results, the timing of Ra-223 administration appeared to have a minimal effect on the survival of patients.

Herein, the number of Ra-223 cycles and PSA level were critical prognostic factors from the initiation of Ra-223 administration. A number of studies have demonstrated that these are significant prognostic factors for patients with BmCRPC (5,8-11,15,16). Ra-223 should be initiated before the PSA levels increase, but other systemic therapies for CRPC should also be initiated during this time. Therefore, the indications for the optimal timing of Ra-223 administration remain unclear. Some studies have suggested that the number of metastatic bone lesions prior to Ra-223 administration are significantly associated with the completion of Ra-223 administration (10,16,17). In the present study, in the univariate analysis, this tended to be associated with a more favorable prognosis. The timing of Ra-223 administration may be considered, depending on the number of bone metastases prior to Ra-223 therapy.

The present study had some limitations associated with its retrospective nature. First, the sample size was small. However, it is a notable finding that the BmCRPC-Ra-223 time, which is a factor not examined in previous studies, reduced the impact of earlier Ra-223 administration on prognosis. Although the present study demonstrated that the impact of an earlier Ra-223 administration was minimal, further studies with greater statistical power are warranted. Second, the completion or non-completion of the Ra-223 regimen affected prognosis in this study, but this had potential risk of immortal time bias (18). Further prospective studies are therefore warranted. Third, half of the cases in the present study had lymph node metastasis, but this may have been missed as our institutions (Ehime University Hospital and the National Hospital Organization Shikoku Cancer Center) did not have prostate-specific membrane antigen (PSMA)-positron emission tomography (PET), which detects where the prostate cancer cells are located in the body. This may have affected treatment outcomes in the present study. Finally, SRE, a critical factor associated with the administration of Ra-223, was not evaluated in the present study as information on SRE was challenging to obtain from the pre-existing medical records. Despite these limitations, the present study provides a novel perspective regarding the timing of Ra-223 administration. However, further large-scale studies are required to confirm the results obtained herein.

In conclusion, the timing of Ra-223 administration did not significantly affect the survival of patients from the initiation of treatment in the present study. Further studies are required to determine the optimal timing for Ra-223 administration.

## Supplementary Material

Survival rates of the patients following radium-223 chloride administration, and the results of univariate and multivariate analyses excluding bone metastasis from the time of castration-resistant prostate cancer radium-223 chloride therapy.

## Figures and Tables

**Table I:** Characteristics of the patients in the present study.

Variable	No. of patients	%
Age, years		
<70	20	39.2
≥70	31	60.8
PS		
0	31	60.8
≥1	20	39.2
No. of bone metastases prior to Ra-223 therapy		
1-5	17	33.3
6-19	17	33.3
≥20	17	33.3
Lymph node metastases prior to Ra-223 therapy		
Yes	22	43.1
No	29	56.9
Use of bone-modifying agents^a^		
Yes	46	90.2
No	5	9.8
EBRT for bone metastatic sites		
Yes	9	17.6
No	42	82.4
Pre-treatment		
Abiraterone	16	31.3
Enzalutamide	26	51.0
Docetaxel	17	33.3
Cabazitaxel	5	9.8
Post-treatment		
Abiraterone	11	21.6
Enzalutamide	19	37.3
Docetaxel	18	35.3
Cabazitaxel	14	27.5
Line of Ra-223 in BmCRPC		
1st	15	29.4
2nd	17	33.3
3rd	12	23.5
4th	4	7.8
5th	3	5.9

^a^Bone-modifying agents include denosumab and zoledronic acid. PS, performance status; Ra-223, radium-223 chloride; BmCRPC, bone metastatic castration-resistant prostate cancer; EBRT external beam radiotherapy.

**Table II:** Survival rates of the patients following radium-223 chloride administration, and the results of univariate and multivariate analyses including the bone metastasis from the time of castration-resistant prostate cancer radium-223 chloride therapy.

	Survival		Univariate analysis			Multivariate analysis		
Variable	1-year (%)	3-year (%)	HR	95% CI	P-value	HR	95% CI	P-value
Age, years			1.47	0.60-3.59	0.40	-	-	-
<70	90	57						
≥70	83	35						
PS			2.79	1.21-6.46	0.02	1.22	0.40-3.73	0.73
0	90	54						
≥1	78	15						
No. of bone metastases prior to Ra-223 therapy			2.51	0.99-6.27	0.06	-	-	-
1-5	94	58						
≥6	81	32						
Lymph node metastases prior to Ra-223 therapy			1.060	0.44-2.55	0.90	-	-	-
Yes	86	42						
No	85	41						
Use of bone-modifying agents^a^			1.69	0.38-7.55	0.49	-	-	-
Yes	86	38						
No	75	75						
EBRT for bone metastatic sites			0.57	0.19-1.70	0.31	-	-	
Yes	60	30						
No	90	44						
Line of Ra-223 in BmCRPC			5.90	1.97-17.63	<0.01	2.67	0.79-9.07	0.11
1-2	87	49						
3-5	74	0						
Completion of Ra-223			42.88	7.91-232.55	<0.01	128.03	10.59-1548.42	<0.01
Yes	97	49						
No	37	0						
PSA levels prior to Ra-223 therapy			6.79	2.47-18.66	<0.01	7.86	2.70-27.24	<0.01
<15.66 ng/ml	100	72						
≥15.66 ng/ml	72	14						
Hemoglobin levels prior to Ra-223 therapy			0.46	0.20-1.08	0.08	-	-	-
<12.3 g/dl	72	31						
≥12.3 g/dl	96	51						
Platelet levels prior to Ra-223 therapy			0.84	0.37-1.94	0.69	-	-	
<21.5x10^4^/µl	84	31						
≥21.5x10^4^/µl	87	53						
ALP levels prior to Ra-223 therapy			1.38	0.59-3.20	0.45		-	
<239 U/l	86	52						
≥239 U/l	84	35						
LDH levels prior to Ra-223 therapy			0.64	0.28-1.48	0.30	-	-	-
<207 U/l	82	37						
≥207 U/l	88	44						
NLR levels prior to Ra-223 therapy			1.72	0.75-3.96	0.20	-	-	-
<2.72	91	50						
≥2.72	80	33						
PLR levels prior to Ra-223 therapy			1.54	0.67-3.55	0.31	-	-	-
<0.88	90	45						
≥0.88	80	42						
BmCRPC-Ra-223 time			1.78	0.76-4.18	0.19	1.29	0.43-3.92	0.65
<1 year	81	51						
≥1 year	89	32						

^a^Bone modifying agents include denosumab and zoledronic acid. PS, performance status; Ra-223, radium-223 chloride; BmCRPC, bone metastatic castration-resistant prostate cancer; EBRT external beam radiotherapy; PSA, prostate-specific antigen; ALP, alkaline phosphatase; LDH, lactate dehydrogenase; NLR, neutrophil-to-lymphocyte ratio; PLR, platelet-to-lymphocyte ratio; BmCRPC-Ra223 time, time from the diagnosis of BmCRPC to the initiation of Ra-223 administration.

## Data Availability

The datasets used and/or analyzed during the current study are available from the corresponding author on reasonable request.
