# Supplementary material for: Impact of timing of radium‑223 administration on the survival of patients with bone metastatic castration‑resistant prostate cancer
Source: Med Int (Lond). 2023 Jul 11;3(4):38. doi: 10.3892/mi.2023.98 (PMC10391592; doi:10.3892/mi.2023.98)
Supplement: Survival rates of the patients following radium-223 chloride administration, and the results of univariate and multivariate analyses excluding bone metastasis from the time of castration-resistant prostate cancer radium-223 chloride therapy. [file Supplementary_Data.pdf]

Table SI. Survival rates of the patients following radium-223 chloride administration, and the results of univariate and multivariate analyses excluding bone metastasis from the time of castration-resistant prostate cancer radium-223 chloride therapy.

	Survival		Univariate analysis			Multivariate analysis		
Variable	1-year (%)	3-year (%)	HR	95% CI	P-value	HR	95% CI	P-value
Age, years			1.47	0.60-3.59	0.40	-	-	-
<70	90	57						
≥70	83	35						
PS			2.79	1.21-6.46	0.02	1.39	0.53-3.65	0.50
0	90	54						
≥1	78	15						
No. of bone metastases prior to Ra-223 therapy			2.51	0.99-6.27	0.06	-	-	-
1-5	94	58						
≥6	81	32						
Lymph node metastases prior to Ra-223 therapy			1.060	0.44-2.55	0.90	-	-	-
Yes	86	42						
No	85	41						
Use of bone-modifying agents[a]			1.69	0.38-7.55	0.49	-	-	-
Yes	86	38						
No	75	75						
EBRT for bone metastatic sites			0.57	0.19-1.70	0.31	-	-	-
Yes	60	30						
No	90	44						
Line of Ra-223 in BmCRPC			5.90	1.97-17.63	<0.01	2.96	1.00-9.27	0.05
1-2	87	49						
3-5	74	0						
Completion of Ra-223			42.88	7.91-232.55	<0.01	154.18	13.40-1773.89	<0.01

Yes	97	49						
No	37	0						
PSA levels prior to Ra-223 therapy			6.79	2.47-18.66	<0.01	8.23	2.40-28.36	<0.01
<15.66ng/ml	100	72						
≥15.66ng/ml	72	14						
Hemoglobin levels prior to Ra-223 therapy			0.46	0.20-1.08	0.08	-	-	-
<12.3 g/dl	72	31						
≥12.3 g/dl	96	51						
Platelet levels prior to Ra-223 therapy			0.84	0.37-1.94	0.69	-	-	-
<21.5x10^4 /μl	84	31						
≥21.5x10^4 /μl	87	53						
ALP levels prior to Ra-223 therapy			1.38	0.59-3.20	0.45	-	-	-
<239 U/l	86	52						
≥239 U/l	84	35						
LDH levels prior to Ra-223 therapy			0.64	0.28-1.48	0.30	-	-	-
<207 U/l	82	37						
≥207 U/l	88	44						
NLR levels prior to Ra-223 therapy			1.72	0.75-3.96	0.20	-	-	-
<2.72	91	50						
≥2.72	80	33						
PLR levels prior to Ra-223 therapy			1.54	0.67-3.55	0.31	-	-	-
<0.88	90	45						
≥0.88	80	42						
BmCRPC-Ra-223 time			1.78	0.76-4.18	0.19	-	-	-
<1 year	81	51						
≥1 year	89	32						

[a]Bone modifying agents include denosumab and zoledronic acid.PS, performance status; Ra-223, radium-223 chloride;BmCRPC, bone metastatic castration-resistant prostate cancer; EBRT external beam radiotherapy;PSA, prostate-specific antigen; ALP, alkaline phosphatase; LDH, lactate dehydrogenase; NLR, neutrophil-to-lymphocyte ratio; PLR, platelet-to-lymphocyte ratio; BmCRPC-Ra223 time, time from the diagnosis of BmCRPC to the initiation of Ra-223 administration.
